# Global Perspectives on Improving Chronic Disease Prevention and Management in Diverse Settings

**DOI:** 10.5888/pcd18.210055

**Published:** 2021-04-08

**Authors:** Collins O. Airhihenbuwa, Tung-Sung Tseng, Victor D. Sutton, LeShawndra Price

**Affiliations:** 1 Health Policy and Behavioral Sciences, School of Public Health, Georgia State University, Atlanta, Georgia; 2 Behavioral and Community Health Sciences Department, School of Public Health, Louisiana State University Health Sciences Center, New Orleans, Louisiana; 3 Office of Preventive Health and Health Equity, Mississippi State Department of Health, Jackson, Mississippi; 4 Office of Research Training and Special Programs, National Institute of Allergy and Infectious Diseases, National Institutes of Health, Bethesda, Maryland

## Abstract

The Centers for Disease Control and Prevention (CDC) define chronic diseases as conditions that last 1 year or more and that require ongoing medical attention or limit activities of daily living, or both (1). Chronic diseases may be influenced by a combination of genetics, lifestyle and social behaviors, health care system factors, community influences, and environmental determinants of health (2). These risk factors often coexist and interact with each other. Therefore, a better understanding of determinants of chronic diseases such as tobacco use, unhealthy eating, and physical inactivity stands to benefit from effective strategies for improving primary, secondary, and tertiary disease prevention and management in diverse global settings (3). Strategies to prevent and manage chronic disease outcomes such as diabetes and cardiovascular diseases (CVDs) have global commonalities (4–7). The impact of chronic diseases is disproportionately evident in Black and Brown communities (8,9). Chronic disease prevention and management typically focus on behavioral interventions such as healthy eating, increased physical activity, and cessation of unhealthy practices such as tobacco and alcohol use (10–15). In 2020, the COVID-19 pandemic added to the fact that chronic diseases disproportionately affect low-resource communities, where many Black and Brown populations live (16,17). COVID-19 demonstrated that chronic disease disparities actually present as preexisting conditions in Black and Brown communities, who are disproportionately affected by COVID-19 outcomes. Although most of the articles in this *Preventing Chronic Disease* (PCD) collection were published before the pandemic, the insights they present, combined with the racial and ethnic data on the burden of COVID-19 thus far, support this reality. Many researchers and public health practitioners often consider the need to sufficiently address the relationships between chronic diseases and social, behavioral, and community factors (18). Global lessons in the prevention and management of chronic diseases, therefore, can help researchers and practitioners benefit from the shared lessons and experience derived from research and interventions conducted in different parts of the world. There are more than 7 billion people worldwide, who speak diverse languages and who have different nationalities, identities, and health systems. Yet, if we share challenges and opportunities for chronic disease prevention and management, many of the global adversities to improving health and well-being can be ameliorated, which is the purpose of this collection. The authors in this collection share lessons that represent experiences in diverse contexts across countries and regions of the world.

## Objective and Themes of the Collection

The goal of the collection is to assemble recently published articles that focus on innovative and effective strategies to improve chronic disease outcomes in diverse populations globally. As efforts to improve global health have accelerated in response to achieving sustainable development goals, chronic disease continues to be a major contributor to poor health outcomes, which often lead to reduction in quality of life, including associated increases in health care costs (19,20). Reducing the burden of chronic diseases remains a global challenge, requiring collaborations across academic disciplines and economic sectors. As discussed in the articles included in this collection, certain individual and societal factors are effective in interventions against chronic diseases. We considered articles that address numerous social and behavioral and risk factors for chronic diseases to offer readers an overview of the similarities and differences that may exist in chronic disease research in diverse global and domestic settings.

This collection consists of 15 articles published over 4 years, from March 2017 to December 2020. This global collection includes articles from North America (United States), South America (Brazil, Mexico, Guatemala), Europe (Spain, Denmark, UK global authors), Asia (Taiwan, Iran, South Korea), Africa (South Africa, Nigeria, Senegal), and Australia. For this collection, the 15 articles were grouped into 2 themes: 1) chronic disease outcomes such as diabetes and CVDs, as well as the impact of COVID-19; and 2) behaviors and strategies (such as healthy food, smoking cessation, breastfeeding, and physical activity) that either prevent or aid in chronic disease management. 

To address chronic disease management and prevention, location matters and is a major factor in achieving research goals. In the United States, barbershops and beauty salons, for example, have been used as sites of health intervention to reach African American communities. Barbershops are considered important cultural venues with convenient community locations for addressing health and social issues that affect the Black community (21). The contribution by Smith et al (22) demonstrated the effectiveness of the Arkansas Minority Barber and Beauty Shop Health Initiative in improving CVD health outcomes of minority populations in the state through health screenings, health education, and disease management. This article describes chronic health conditions that presented during screening at a barber shop or beauty salon and the impact of a health education promotion campaign at this location. In addition, it showed how medical referrals and participant follow-ups can be integrated into screening initiatives based in barber shops and beauty salons. Specific chronic diseases included diabetes, CVD, and obesity.

Diabetes is associated with illness and premature death and affects an estimated 285 million people, which corresponds to 6.4% of the world’s adult population (23). Globally, type 2 diabetes is a growing public health challenge, and public health and health care systems play important roles in prevention and management of complications related to both type 1 and type 2 diabetes (23). Some programs, such as weight or diet management, physical activity, hemoglobin A_1c_ control, and diabetic retinopathy screening, are useful approaches for diabetes prevention and management. The article by Mendoza-Herrera et al (24) presents a low-cost and easy-to-apply new screening tool to detect people who are at high risk for diabetic retinopathy in low-income Mexican communities. This noninvasive screening tool for diabetic retinopathy could be used by nonspecialized health personnel in low-income communities. The screening tool requires the assessment of glycemia, blood pressure, and information related to diabetes and physical activity recommendations. From a clinical or health care perspective, many diabetes management programs provide short-term or long-term quality diabetes care. In a systematic review of the literature, pay-for-performance (P4P) or value-based purchasing programs were shown, across many countries, to contribute to the efficient delivery of long-term, multidisciplinary diabetes management behaviors (25). Hsieh et al (26) evaluated the effects of diabetes management on risks of cancer incidence and mortality among patients with type 2 diabetes through a diabetes P4P program in Taiwan. This study showed that diabetes P4P programs reduced risks of all-cause mortality and competing causes of death due to cancer-specific and diabetes-related mortality among patients with type 2 diabetes. Other studies focusing on chronic disease management programs (CDMPs) have incorporated health coaching into their programs, as was done at Royal North Shore Hospital in Sydney, Australia, in 2013 (27). This study assessed changes in patients’ general knowledge of diabetes, self-reported health status, diabetes distress, body mass index (BMI), and glycemic control after enrollment in a face-to-face CDMP group health coaching session (with telephone follow-up), compared with participation in telephone-only health coaching, during a 12-month period. 

In addition to addressing chronic diseases like diabetes, some articles also addressed risk factors such as obesity. Obesity is not only a chronic disease but also a risk factor for other diseases, including heart disease, hypertension, and some cancers. According to CDC, from 1999–2000 through 2017–2018, the prevalence of obesity in the US increased from 30.5% to 42.4%, and the prevalence of severe obesity increased from 4.7% to 9.2% (28). The association between obesity and chronic disease risk factors is complex and may vary by population. Risk factors or risk situations include those addressed in this collection, including food swamps, poor eating, and physical inactivity. The article by Petersen et al (29) introduced efforts by CDC’s Division of Nutrition, Physical Activity, and Obesity (DNPAO) to address related racial and ethnic disparities. For example, DNPAO competitively funds 16 state health departments (or similar entities), 15 land grant colleges and universities, and 31 community-focused grantees to work with multiple sectors and coalitions to prioritize and implement best practices for increasing healthy eating and active living to prevent obesity and other chronic diseases. In addition, 2 public health practice programs, the Racial and Ethnic Approaches to Community Health program and the High Obesity Program, have had success in reducing risk factors for obesity in target populations with the highest disparities. For surveillance of obesity prevalence, DNPAO has published state-specific obesity maps using self-reported data of height and weight from the Behavioral Risk Factor Surveillance System since 1999. These maps have shown that obesity prevalence among adults remains high across the country, year by year. Many research efforts to prevent obesity and other chronic disease risks exist, as presented in the second theme of this collection.

Evidence shows that physical activity reliably protects against CVDs. In the article by Modesto et al (30), the team analyzed whether an individualized, prescribed intervention that consists of walking in the park would assist in improving cardiovascular health among participants. A total of 1,466 adults aged 40 to 80 were initially enrolled into the study, and 152 participants completed the full postintervention reevaluation and followed the prescribed regimens within 3 to 6 months. Among those who completed the full walk-in-park intervention, significant changes were found in BMI, waist circumference, and systolic blood pressure. Diastolic blood pressure, blood glucose, and total cholesterol remained unchanged. The article by López-Bueno and colleagues (31) describes a study designed to analyze whether levels of physical activity were associated with higher odds of common chronic conditions within the Spanish workforce. Using data from the Spanish National Health Survey 2017 (N = 9,695), López-Bueno examined differences in the 6 most prevalent chronic conditions among a workforce that engaged in physical activity. Results indicated that participants who performed less physical activity per week were significantly more likely to experience chronic conditions than those who performed more. In a related study on the benefits of leisure time physical activity, Sturm and Cohen (32) found that not having free time was not enough to explain physical inactivity in the study population. In reviewing data that covered a 3-year period (2014–2016) for more than 32,000 people aged 15 years or older, the authors examined activity types and levels in places where participants learned, prayed, or worked. They concluded that motivation to exercise was an important factor to consider even for those who have free time. As more Americans continue to be physically inactive, the authors suggested that making spaces for physical activity be more inviting and engaging is important to increasing the level of physical activities for adults. 

Just as increasing physical activity is important, so too is healthy nutrition. Maxwell et al (33) adapted a British healthy eating intervention for elementary school children in an under-resourced setting in South Los Angeles to promote healthier eating and improve children’s long-term health outcomes. This intervention was developed in collaboration with YMCA with the goal of promoting eating vegetables among young people. This intervention, based on the British-developed Tiny Tastes program, was designed to expose young people to small portions of vegetables as a way of encouraging them to adopt the habit of eating vegetables. Fifty children aged 7 to 12 years who attended a summer camp were repeatedly exposed to initially disliked vegetables, daily, over a 2-week period. Follow-up assessments were conducted immediately after the last exposure (2 weeks) and after 14 additional days of nonexposure (4-weeks follow up). Findings showed a significant increase in children’s propensity toward initially disliked vegetables to which they were repeatedly exposed but not to vegetables to which they did not receive repeated exposure. Results of the study demonstrate that repeated vegetable tasting strategies offered in community settings may be a practical, low-cost, easy-to-implement strategy for health promotion, decreasing prevalence of chronic diseases, and subsequently improving population health in under-resourced communities.

Public health and medical researchers use body mass index (BMI) to measure risk for chronic diseases. However, questions remain as to whether the standard BMI measure accurately captures variance that may exist in different racial and ethnic populations. The contribution by Darbandi et al (34) is a systematic meta-analysis of studies that use BMI to predict CVD. The analysis included adults aged 18 or older in cross-sectional and prospective cohort studies. The studies were conducted between 1996 and 2019 in 15 countries. Of all the studied indexes, it appears that BMI, waist circumference, and waist-to-hip ratio all presented reliable predictive power for risk of CVD. However, waist-to-hip ratio indexes presented stronger predictive power. In another study on measurement of risks, Lee et al (35) proposed a cumulative social risk composite score that provides specificity in predicting CVD beyond the traditional Framingham risk score. Their analysis used a nationally representative group of South Korean adults aged 19 or older with CVD, using the cumulative social risk measure (which includes income, education, and marital status).

Collective multidisciplinary approaches are needed to address risk behaviors as a life course determinant of chronic diseases. For example, the need to continue to address healthy diet at an early age is a strategy to reduce obesity in childhood. Still, early age healthy eating can produce positive health benefits over one’s lifespan. The article by Garvin et al (36) showed, through an evaluation project of the National Early Childhood and Education Learning Collaborative (ECELC) programs, that 10 states were successful in increasing healthy eating habits of young people. The ECELC programs demonstrated the benefits and merits of a multidisciplinary approach to implementation and evaluation of a public health intervention program. The authors evaluated a multidisciplinary and multisectoral partnership strategy to promote healthy eating and increased physical activity in early childhood. They concluded that this type of collective approach offers an opportunity for an early intervention to prevent chronic diseases. Another article assessed the nutritional challenges of an unhealthy food environment in Guatemala, particularly in locations where schools are swamped with unhealthy foods. Food swamps, as opposed to food deserts, have mostly been reported in the Global North as a result of the proliferation of fast foods in low-income communities. The article by Chew et al (37) is one of the few that have reported on food swamps in the Global South as evidence of the high concentration of fast food outlets in low-income locations around schools. The article demonstrates how food swamps are a global health problem and pose a particular threat of adopting unhealthy food practices in communities. As we address unhealthy behaviors, it is important to learn from those who have changed their behaviors from unhealthy to healthy behaviors. The article by Murphy-Hoefer et al (38) reported lessons learned from people who have been successful in discontinuing smoking. The lessons of former smokers may hold promise for smoking cessation efforts globally, particularly as we begin to address the epidemic of vaping.

In this collection, 1 article shows the intersection of chronic diseases and infectious diseases such as COVID-19. Black and Brown populations are disproportionately affected by both chronic diseases and COVID-19. The article by Airhihenbuwa et al (39) makes the point that for historically disempowered communities to benefit from public health interventions, both individual and structural factors must be equally valued in communicating about risks and mitigation. The article addresses the fact that COVID-19 messaging that focuses on individual risk behaviors, such as hand washing and physical distancing, may not fully account for contexts in which congested housing and public-facing jobs structurally make individually focused messages untenable. Equally addressing individual and collective risks allows us to address the structural and social determinants (eg, food access, job availability, housing conditions) that influence risks and vulnerability for COVID-19 and chronic diseases. To address the contexts, the PEN-3 cultural model was presented as an example.

## The Future of Global Chronic Disease Research and Action

The articles in this collection reflect a common problem and solution for chronic diseases globally. The strategies offered in one context hold promise for others. The articles demonstrate the need to balance individual-based prevention and management efforts (like healthy eating and increased physical activity) with environmentally based strategies like food swamps or food deserts. If we are going to be successful in improving health globally, voices from the global community must be heard and incorporated into the interventions to have relevant, appropriate, and optimal outcomes for different regions of the world (40). Environmental and systemic risks should be considered as ways of providing contexts for individual risks. For example, COVID-19’s disproportionate impact on those with chronic diseases in Black and Brown communities is well documented. In PCD in 2020, these articles shed light on the tasks ahead, which is to address the historical contexts of racial inequities and to promote social justice as global health.

At the global level, chronic disease is commonly referred to as, or used interchangeably with, noncommunicable disease (NCD). NCD has gained increased attention as its burden has outstripped that of infectious/communicable diseases in the Global South. The rate of increase in the burden of NCDs is unprecedented and precipitated the 2018 United Nations high-level commission to frame a global response to NCDs (41). The focus of the 2018 meeting was not only to address risk behaviors but also to highlight structural levers of change, therefore highlighting the urgency for countries to prioritize NCD reduction goals and objectives to have both health and economic benefits. Prioritizing NCDs should be considered an investment with economic gains that can be calculated in dollars saved, productivity increased, and overall economic growth (42). A global urgency exists for chronic disease and other health intervention strategies to advance health equity for Black and Brown populations, which can be done only when we collectively address structures and systems, such as place and group status, rather than focusing only on individuals (43). For example, there is a renewed call to decolonize global health (44). The renewed demand for decolonization has been led mostly by public health students, notably at Harvard and Duke Universities. The demand to decolonize global health has been further reignited in large part by the Black Lives Matter movement, whose goal to address structural and systemic racism became a global movement following the killing of George Floyd, Breonna Taylor, and others. The future of reducing chronic diseases globally must therefore be connected with collective efforts to address global health inequities.

## Conclusion

The articles presented in this collection suggest that innovation in evidence-based approaches is essential to improving population-based health strategies for chronic disease prevention and management. Collective sharing and learning across countries and regions is needed. Our approaches should be anchored in community-engaged, multidisciplinary, and multisectoral relationships to prevent and manage chronic diseases globally. Committing to this global North/South sharing and learning must be strengthened and expanded to inform policy, transform systems, and contextualize strategies so that interventions are responsive to both individual and structural changes and sustainability.

Global health begins with local health. Each article in this collection reflects chronic disease issues in local settings with global relevance and lessons. These articles clearly show that the local is part of the global. Preventing and managing chronic diseases reinforce the value that individual experiences hold lessons in both promise and challenges that can be shared globally. Important and timely as the articles included in this collection are, global health should help advance strategies that address structural and systemic determinants of chronic diseases so that individual behavior change can be sustainable. Although we have learned much from these articles, what is needed for the future is research to address more of the structural levers to preventing and managing chronic diseases to improve quality of life where people work, live, play, pray, and learn. Thus, helping to change individual behaviors relative to eating, physical activity, and sleep are important but should be better understood within the contexts of the condition in the same: where people live (housing conditions), work (if they have jobs), play (if they have space), pray (to develop resilience), and learn (if they have access to quality education).

## References

[1] Centers for Disease Control and Prevention. About chronic diseases. https://www.cdc.gov/chronicdisease/about/index.htm. Accessed March 3, 2021.

[2] Cockerham WC , Hamby BW , Oates GR . The social determinants of chronic disease. Am J Prev Med 2017;52(S1):S5-S12. 10.1016/j.amepre.2016.09.010 PMC5328595 27989293

[3] Magnusson RS . Global health governance and the challenge of chronic, non-communicable disease. J Law Med Ethics 2010;38(3):490–507. 10.1111/j.1748-720X.2010.00508.x 20880237

[4] Aifah A , Iwelunmor J , Akwanalo C , Allison J , Amberbir A , Asante KP , The Kathmandu Declaration on Global CVD/Hypertension Research and Implementation Science: a framework to advance implementation research for cardiovascular and other noncommunicable diseases in low- and middle-income countries. Glob Heart 2019;14(2):103–7. 10.1016/j.gheart.2019.05.006 31324363 PMC7341552

[5] Al-Lawati JA . Diabetes mellitus: a local and global public health emergency! Oman Med J 2017;32(3):177–9. 10.5001/omj.2017.34 28584596 PMC5447787

[6] Bansilal S , Castellano JM , Fuster V . Global burden of CVD: focus on secondary prevention of cardiovascular disease. Int J Cardiol 2015;201(Suppl 1):S1–7. 10.1016/S0167-5273(15)31026-3 26747389

[7] Smith SL , Gorantla R . Analysing the global health agenda: a comparison of priority for diabetes and oral diseases. Glob Public Health 2020;1–15. 10.1080/17441692.2020.1814834 32903145

[8] Al Kibria GM . Racial/ethnic disparities in prevalence, treatment, and control of hypertension among US adults following application of the 2017 American College of Cardiology/American Heart Association guideline. Prev Med Rep 2019;14:100850. 10.1016/j.pmedr.2019.100850 31061780 PMC6488531

[9] Price JH , Khubchandani J , McKinney M , Braun R . Racial/ethnic disparities in chronic diseases of youths and access to health care in the United States. BioMed Res Int 2013;2013:787616. 10.1155/2013/787616 24175301 PMC3794652

[10] Cannata F , Vadalà G , Russo F , Papalia R , Napoli N , Pozzilli P . Beneficial effects of physical activity in diabetic patients. J Funct Morphol Kinesiol 2020;5(3):70. 10.3390/jfmk5030070 33467285 PMC7739324

[11] Krist AH , Davidson KW , Mangione CM , Barry MJ , Cabana M , Caughey AB , ; US Preventive Services Task Force. Interventions for tobacco smoking cessation in adults, including pregnant persons: US Preventive Services Task Force Recommendation statement. JAMA 2021;325(3):265–79. 10.1001/jama.2020.25019 33464343

[12] Lee JA , Choi M , Lee SA , Jiang N . Effective behavioral intervention strategies using mobile health applications for chronic disease management: a systematic review. BMC Med Inform Decis Mak 2018;18(1):12. 10.1186/s12911-018-0591-0 29458358 PMC5819153

[13] Ricci C , Wood A , Muller D , Gunter MJ , Agudo A , Boeing H , Alcohol intake in relation to non-fatal and fatal coronary heart disease and stroke: EPIC-CVD case-cohort study. BMJ 2018;361:k934. 10.1136/bmj.k934 29844013 PMC5972779

[14] Tseng TS , Moody-Thomas S , Horswell R , Yi Y , Celestin MD , Jones KD . Using a health informatics system to assess effect of a federal cigarette tax increase on readiness to quit among low-income smokers, Louisiana, 2009. Prev Chronic Dis 2014;11:130203. 10.5888/pcd11.130203 24698530 PMC3976230

[15] Yan X , Han X , Wu C , Shang X , Zhang L , He M . Effect of physical activity on reducing the risk of diabetic retinopathy progression: 10-year prospective findings from the 45 and Up Study. PLoS One 2021;16(1):e0239214. 10.1371/journal.pone.0239214 33444338 PMC7808642

[16] Doshi RP , Aseltine RH Jr , Sabina AB , Graham GN . Racial and ethnic disparities in preventable hospitalizations for chronic disease: prevalence and risk factors. J Racial Ethn Health Disparities 2017;4(6):1100–6. 10.1007/s40615-016-0315-z 27924622

[17] Ivers LC , Walton DA . COVID-19: global health equity in pandemic response. Am J Trop Med Hyg 2020;102(6):1149–50. 10.4269/ajtmh.20-0260 32297589 PMC7253101

[18] Jack L Jr . Disseminating timely peer-reviewed content in 2020: COVID-19 and chronic disease, public health and pharmacy, eliminating health disparities, global health, and student research. Prev Chronic Dis 2020;17:200447. 10.5888/pcd17.200447 32975506 PMC7553225

[19] Van Alsten SC , Harris JK . Cost-related nonadherence and mortality in patients with chronic disease: a multiyear investigation, National Health Interview Survey, 2000–2014. Prev Chronic Dis 2020;17:200244. 10.5888/pcd17.200244 33274701 PMC7735485

[20] Wimalawansa SJ . Public health interventions for chronic diseases: cost-benefit modelizations for eradicating chronic kidney disease of multifactorial origin (CKDmfo/ CKDu) from tropical countries. Heliyon 2019;5(10):e02309. 10.1016/j.heliyon.2019.e02309 31720441 PMC6838989

[21] Luque JS , Ross L , Gwede CK . Qualitative systematic review of barber-administered health education, promotion, screening and outreach programs in African-American communities. J Community Health 2014;39(1):181–90. 10.1007/s10900-013-9744-3 23913106 PMC3947222

[22] Smith C , Porter A , Biddle J , Balamurugan A , Smith M . The Arkansas Minority Barber and Beauty Shop Health Initiative: meeting people where they are. Prev Chronic Dis 2020;17:200277. 10.5888/pcd17.200277 33274699 PMC7735488

[23] Bergman M , Buysschaert M , Schwarz PE , Albright A , Narayan KV , Yach D . Diabetes prevention: global health policy and perspectives from the ground. Diabetes Manag (Lond) 2012;2(4):309–21. 10.2217/dmt.12.34 26339296 PMC4556601

[24] Mendoza-Herrera K , Quezada AD , Pedroza-Tobias A , Hernandez-Alcaraz C , Fromow-Guerra J , Barquera S . A diabetic retinopathy screening tool for low-income adults in Mexico. Prev Chronic Dis 2017;14:170157. 10.5888/pcd14.170157 PMC5645201 29023230

[25] de Bruin SR , Baan CA , Struijs JN . Pay-for-performance in disease management: a systematic review of the literature. BMC Health Serv Res 2011;11(1):272. 10.1186/1472-6963-11-272 21999234 PMC3218039

[26] Hsieh HM , He JS , Shin SJ , Chiu HC , Lee CT . A diabetes pay-for-performance program and risks of cancer incidence and death in patients with type 2 diabetes in Taiwan. Prev Chronic Dis 2017;14:170012. 10.5888/pcd14.170012 28981404 PMC5645199

[27] Delaney G , Newlyn N , Pamplona E , Hocking SL , Glastras SJ , McGrath RT , Identification of patients with diabetes who benefit most from a health coaching program in chronic disease management, Sydney, Australia, 2013. Prev Chronic Dis 2017;14:160504. 10.5888/pcd14.160504 28253473 PMC5338599

[28] Hales CM , Fryar CD , Ogden CL . Prevalence of obesity and severe obesity among adults: United States, 2017–2018. NCHS Data Brief 2020;360:1–8. 32487284

[29] Petersen R , Pan L , Blanck HM . Racial and ethnic disparities in adult obesity in the United States: CDC’s tracking to inform state and local action. Prev Chronic Dis 2019;16:180579. 10.5888/pcd16.180579 30974071 PMC6464044

[30] Modesto B , Bartholomeu T , Basso L , Costa L , Tinucci T , Forjaz C . Effects of a real-life park-based physical activity interventional program on cardiovascular risk and physical fitness. Prev Chronic Dis 2021;18:200115. 10.5888/pcd18.200115 PMC7938965 33630729

[31] López-Bueno R , Bláfoss R , Calatayud J , López-Sánchez GF , Smith L , Andersen LL , Association between physical activity and odds of chronic conditions among workers in Spain. Prev Chronic Dis 2020;17:200105. 10.5888/pcd17.200105 33034558 PMC7553219

[32] Sturm R , Cohen DA . Free time and physical activity among Americans 15 years or older: cross-sectional analysis of the American Time Use Survey. Prev Chronic Dis 2019;16:190017. 10.5888/pcd16.190017 31560643 PMC6795070

[33] Maxwell AE , Castillo L , Arce AA , De Anda T , Martins D , McCarthy WJ . Eating veggies is fun! An implementation pilot study in partnership with a YMCA in South Los Angeles. Prev Chronic Dis 2018;15:180150. 10.5888/pcd15.180150 30388069 PMC6219845

[34] Darbandi M , Pasdar Y , Moradi S , Mohamed HJJ , Hamzeh B , Salimi Y . Discriminatory capacity of anthropometric indices for cardiovascular disease in adults: a systematic review and meta-analyis. Prev Chronic Dis 2020;17:200112. 10.5888/pcd17.200112 33092686 PMC7587303

[35] Lee H , Kang AW , Lee H , Cha Y , Operario D . Cumulative social risk and cardiovascular disease among adults in South Korea: a cross-sectional analysis of a nationally representative sample. Prev Chronic Dis 2020;17: 190382. 10.5888/pcd17.190382 PMC7279061 32463785

[36] Garvin TM , Weissenburger-Moser Boyd L , Chiappone A , Blaser C , Story M , Gertel-Rosenberg A , Multisector approach to improve healthy eating and physical activity policies and practices in early care and education programs: The National Early Care and Education Learning Collaboratives Project, 2013–2017. Prev Chronic Dis 2019;16:180582. 10.5888/pcd16.180582 31344337 PMC6716417

[37] Chew A , Moran A , Barnoya J . Food swamps surrounding schools in three areas of Guatemala. Prev Chronic Dis 2020;17:200029. 10.5888/pcd17.200029 32762810 PMC7417024

[38] Murphy-Hoefer R , Davis KC , Beistle D , King BA , Duke J , Rodes R , Impact of the Tips From Former Smokers Campaign on Population-Level Smoking Cessation, 2012–2015. Prev Chronic Dis 2018;15:180051. 10.5888/pcd15.180051 29862960 PMC5985905

[39] Airhihenbuwa CO , Iwelunmor J , Munodawafa D , Ford CL , Oni T , Agyemang C , Culture matters in communicating the global response to COVID-19. Prev Chronic Dis 2020;17:200245. 10.5888/pcd17.200245 32644918 PMC7367065

[40] Ford CL , Airhihenbuwa CO . Commentary: just what is critical race theory and what’s it doing in a progressive field like public health? Ethn Dis 2018;28(Suppl 1):223–30. 10.18865/ed.28.S1.223 30116090 PMC6092167

[41] Heller O , Somerville C , Suggs LS , Lachat S , Piper J , Aya Pastrana N , The process of prioritization of non-communicable diseases in the global health policy arena. Health Policy Plan 2019;34(5):370–83. 10.1093/heapol/czz043 31199439 PMC6736081

[42] Saving lives, spending less: a strategic response to noncommunicable diseases. Geneva (CH): World Health Organization; 2018.

[43] Brown AF , Ma GX , Miranda J , Eng E , Castille D , Brockie T , Structural interventions to reduce and eliminate health disparities. Am J Public Health 2019;109(S1):S72–8. 10.2105/AJPH.2018.304844 30699019 PMC6356131

[44] Büyüm AM , Kenney C , Koris A , Mkumba L , Raveendran Y . Decolonising global health: if not now, when? BMJ Glob Health 2020;5(8):e003394. 10.1136/bmjgh-2020-003394 32759186 PMC7409954

